# Supplementation with a Whey Protein Concentrate Enriched in Bovine Milk Exosomes Improves Longitudinal Growth and Supports Bone Health During Catch-Up Growth in Rats

**DOI:** 10.3390/nu16223814

**Published:** 2024-11-07

**Authors:** Jorge García-Martínez, Rafael Salto, María D. Girón, Íñigo M. Pérez-Castillo, Pilar Bueno Vargas, Jose D. Vílchez, Azahara Linares-Pérez, Manuel Manzano, María T. García-Córcoles, Ricardo Rueda, José M. López-Pedrosa

**Affiliations:** 1 Abbott Nutrition R&D, Abbott Laboratories, 18004 Granada, Spain; inigomaria.perez@abbott.com (Í.M.P.-C.); pilar.bueno@abbott.com (P.B.V.); manuel.manzano@abbott.com (M.M.); mariateresa.garcia@abbott.com (M.T.G.-C.); ricardo.rueda@abbott.com (R.R.); jose.m.lopez@abbott.com (J.M.L.-P.); 2 Department of Biochemistry and Molecular Biology II, School of Pharmacy, University of Granada, Campus de Cartuja, 18071 Granada, Spain; rsalto@ugr.es (R.S.); mgiron@ugr.es (M.D.G.); e.damaso@go.ugr.es (J.D.V.); azaharalinares@ugr.es (A.L.-P.)

**Keywords:** exosomes, bovine milk, extracellular vesicles, catch-up growth, linear growth, preclinical, chondrocyte, bone microstructure, growth plate, malnutrition

## Abstract

Background: Undernutrition impairs linear growth while restoration of nutritional provisions leads to accelerated growth patterns. However, the composition of the nutrition provided is key to facilitating effective catch-up growth without compromising bone quantity, quality, and long-term health. Methods: We evaluated the role of a whey protein concentrate enriched in bovine milk exosomes (BMEs) in modulating the proliferative properties of human chondrocytes in vitro and studied how these effects might impact bone quantity and quality measured as longitudinal tibia growth, bone mineral content (BMC) and density (BMD), and trabecular micro-CT parameters in stunted rats during catch-up growth. Results: BMEs promoted proliferation in C28/I2 human chondrocytes mediated by mTOR-Akt signaling. In a stunting rat model, two-week supplementation with BMEs during refeeding was associated with improved tibia BMD, trabecular microstructure (trabecular number (Tb. N.) and space (Tb. Sp.)), and a more active growth plate (higher volume, surface, and thickness) compared to non-supplemented stunted rats. Positive effects on physis translated to significantly longer tibias without compromising bone quality when extending the refeeding period for another two weeks. Conclusions: Overall, BME supplementation positively contributed to longitudinal bone growth and improved bone quantity and quality during catch-up growth. These findings might be relevant for improving diets aimed at addressing the nutritional needs of children undergoing undernutrition during early life.

## 1. Introduction

Linear growth, commonly expressed as length/height-for-age z-scores (LAZ and HAZ) [1], is a major contributor to infant development [2]. Stunting, defined as HAZ < 2 standard deviations (SDs) from the global median [1], is estimated to impact 29.1% of all children < 5 years old worldwide [3] and is associated with a higher risk of all-cause and cause-specific mortality (i.e., diarrheal disease and infection) [4]. Since most linear growth faltering already occurs by the age of 2 years old [5], the early stages of life represent a window of opportunity for interventions aimed at preventing stunting and supporting healthy infant development. Unfortunately, there is a dearth of effective interventions aimed at improving linear growth and preventing stunting during this phase [5].

Genome-wide-data analyses suggest that common single-nucleotide polymorphisms (SNPs) only account for ≈50% of human height variation [6], while the rest is impacted by environmental factors, most notably nutrition [7]. In fact, food restriction can severely compromise linear growth [8], and restoration of sufficient nutritional provisions can lead to accelerated growth patterns in a process termed “catch-up growth” [9]. However, accelerated infant growth fueled by unhealthy nutrition (i.e., diets rich in high-glycemic-index carbohydrates) has been shown to be associated with a “thrifty phenotype” linked to a greater risk of metabolic diseases such as obesity [10,11], which highlights the need for nutritional interventions designed for tackling growth faltering in children without compromising long-term health.

Although governed at the endocrine level, the process of longitudinal bone growth is intrinsic to the growth plate (physis) [12]. The structure of the growth plate briefly consists of chondrocytes suspended in a collagen matrix, which undergo coordinated phases of maturation to eventually die, leaving behind a calcified matrix [13,14]. This process of chondrocyte maturation is termed endochondral ossification and eventually results in vascularization and bone elongation (reviewed in detail in Ref. [13]). Final height is attained due to the closure of the growth plate following endochondral ossification, but dietary restriction can arrest this process [15]. Specifically, undifferentiated chondrocytes become dormant under growth-inhibiting conditions such as nutrient deprivation, but preserve their proliferative potential until these conditions are resolved, a process termed “delayed growth plate senescence” [16]. Catch-up growth induced by renewal of food supply precipitates growth plate senescence [16]; however, the loss of the proliferative potential of chondrocytes during this process might be associated with incomplete catch-up [17]. Accelerated growth patterns might also result in impaired bone quality [18], which is speculated to be associated with an increased risk of bone fractures later in life [19,20]. Crucially, different nutritional components such as proteins [21], lipids [22], and carbohydrates [23] have been documented to differentially impact features of growth plate development and bone health, such as mineral density, mineral content, and/or microstructure during catch-up growth. Further, recent research has provided compelling direct evidence on the role of nutrient unavailability in growth plate activity, and signaling mechanisms involved in the rescue of longitudinal bone growth once sufficient nutrient provisions are restored [24]. Altogether, these studies highlight the need for evaluating novel nutritional interventions aimed to support effective catch-up growth associated with healthier bone phenotypes and elucidate the mechanisms involved.

A nutritional component that has witnessed breakthrough progress in recent years is bovine milk exosomes. These consist of a subset of milk-derived extracellular vesicles (sized around 20–200 nm) containing different membrane components and cargos, namely lipids, proteins, and nucleic acids, which have been proposed to exert beneficial effects at multiple levels including musculoskeletal health, growth, and development among others [25]. MiRNAs abundantly expressed in bovine milk exosomes have been speculated to promote postnatal growth through Mechanistic Target of Rapamycin (mTOR), a master regulator of nutrient signaling and cellular growth [26,27], as well as through Insulin-like Growth Factor 1 (IGF-1) signaling [28,29]. Particularly, the mTOR-Akt pathway has a central role in the coordination of chondrocyte proliferation and differentiation. At early stages of the process, mTOR activation is needed to increase the population of active chondrocytes, while at later stages mTOR activity remains essential to modulate chondrocyte final differentiation [30]. Interestingly, bovine milk exosome administration has been documented to enhance osteoblast differentiation and reduce bone resorption in healthy young adult mice (10–12 weeks old), thus indicating a potential positive role in bone formation [31,32]. Moreover, ensuing studies reported osteoprotective effects of bovine milk exosomes in different preclinical models of bone loss [33,34,35]. Lastly, Go et al. reported data from a preclinical study where six-week-old Sprague–Dawley rats were orally administered 50 mg/kg/day of bovine milk exosomes for 14 days. The authors concluded that bovine milk exosome administration was safe and associated with promoted longitudinal bone growth and increased tibia cortical and trabecular bone mineral density compared to the control animals (PBS) [36]. However, no study to date has examined the effects of bovine milk exosomes on chondrocyte proliferation—considered the primary factor contributing to longitudinal bone growth in humans [37]—and the application of oral bovine milk exosome supplementation to support bone health and development during catch-up growth remains unexplored.

In the present research, we aimed to evaluate the effects of a whey protein concentrate enriched in bovine milk exosomes (BMEs) on the proliferative properties of human chondrocytes in vitro to later explore how these findings may translate to a model of diet-induced catch-up growth in stunted rats to improve longitudinal growth and bone health outcomes. Our research indicates that incubation with BMEs enhances the proliferative activity of human chondrocytes in vitro through the activation of the mTOR-Akt pathway. Additionally, a two-week regimen of BME supplementation was correlated with increased growth plate activity and improved trabecular microstructure in the tibia of stunted rats during catch-up growth, suggesting greater potential for longitudinal bone growth and improved bone quality compared to control diet consumption. Moreover, the observed positive impact on growth plate activity translated to significantly longer tibias and accelerated growth rate following an extended two-week BME supplementation period. This occurred without compromising bone densitometry or micro-CT parameters, nor was it associated with excess fat gain.

## 2. Materials and Methods

### 2.1. Bovine Milk Exosome Enrichment Methods and Supplementation of the Experimental Diet

To date there is no available method that permits the obtention of 100% pure bovine milk exosomes scalable at the manufacturing level, thus we aimed to obtain a whey protein concentrate enriched in bovine milk exosomes. To this end, bovine milk exosomes were isolated from commercial cheese whey using multiple ceramic filtration steps in tandem. Briefly, a first permeate was obtained through ultrafiltration using a 1.4 µm size pore filter, and a second retentate was retrieved following ultrafiltration with a 0.14 µm size pore filter. The resulting retentate was diluted in water to be later diafiltered and diluted again in serial steps using a 10 kDa membrane. The collected concentrate was pasteurized at 70 °C for 15 s to ensure microbiological stability. The pasteurized concentrate was then evaporated at 65 °C until the solids content reached 17–18%. The resulting stream was finally spray-dried at 185 °C/85 °C to obtain the whey protein concentrate enriched in bovine milk exosomes (BMEs) used in in vitro experiments and to supplement the experimental diet. Western blot analyses were performed to characterize bovine milk exosomes in the resulting concentrate based on the presence of TSG101 and CD9, two known exosome protein markers [38,39], and exosome content was evaluated through interferometric and fluorescence detection analyses in the commercial cheese whey and the resulting BMEs using an ExoView R200^TM^ analyzer (Unchained Labs, Pleasanton, CA, USA) and ExoFlex^TM^ chips (Unchained Labs, Pleasanton, CA, USA) (Figure S1). ExoView methods are detailed in the Supplementary Materials. Finally, an adapted version of the standardized rodent maintenance diet “AIN93M” (composition presented in Table S1) was supplemented with the BMEs for in vivo studies to achieve bovine milk exosome enrichment compared to the non-supplemented diet, and exosome content analysis was performed through ExoView both in the control and experimental diets (Figure S2).

### 2.2. In Vitro Research and Experimental Design

All in vitro experiments were conducted in immortalized C28/I2 human chondrocytes (#SCC043, Sigma-Aldrich, Madrid, Spain) grown to 70–90% confluence in Dulbecco’s modified Eagle’s medium (DMEM), containing 10% fetal bovine serum, 100 U/mL of penicillin, and 100 µg/mL of streptomycin plus 2 mM of glutamine in a 5% CO_2_ atmosphere at 37 °C. All in vitro experiments were conducted in duplicate, and biological replicates (n) are specified in each experiment.

### 2.3. Cell Growth Test

Chondrocyte growth was tested using a tetrazolium dye (MTT) reduction assay following treatment with increasing amounts (0, 5, 15, 30, and 50 µg/mL final protein concentrations) of BMEs in growth medium for the indicated period (*n* = 6). Then, 50 µg/mL of MTT was added to the culture medium and incubated at 37 °C for 30 min. Following incubation, the MTT-containing medium was removed and acidic isopropanol (40 mM of HCl in isopropanol) was added to dissolve the resulting formazan crystals. Optical densities (OD) were measured at 570 nm using an absorbance microplate reader. Cell growth (%) was normalized based on OD values in non-treated cells.

As a control, proliferation assays were also carried out using sonicated BMEs (a process known to damage exosome structure and degrade exosomal miRNA, thus disrupting exosome uptake and biological function [40]), and the MTT test was performed under the same conditions as above (*n* = 4). Lastly, the MTT test was performed under inhibited activation of mTOR and Akt through preincubating either with 25 µM of rapamycin (mTOR inhibitor, Sigma-Aldrich, St. Louis, MO, USA) or 50 µM of LY294002 (PI3K-Akt inhibitor, Sigma-Aldrich, St. Louis, MO, USA), and adding 30 µg/mL of BMEs (24 h incubation) to evaluate the signaling mechanisms involved (*n* = 6).

### 2.4. Cell Cycle Analysis

Chondrocytes were incubated with 0 or 15 µg/mL of BMEs for 24 h to be later dissociated through trypsinization and centrifugation at 200× *g* for 5 min at room temperature (*n* = 10). The resulting pellets were re-suspended in 500 µL of PBS and centrifuged again at 200× *g* for 1 min. The PBS was removed and the cells were fixed by adding 1 mL of ice-cold 70% ethanol and incubating for 1 h at −20 °C. The fixed cells were collected through centrifugation at 400× *g* for 5 min, and the supernatant was removed. The cells were washed in 1 mL of PBS and centrifuged at 400× *g* for 1 min. Total DNA staining was achieved by re-suspending the pellets in 500 µL of a staining solution consisting of 200 mg of propidium iodide in 10 mL of PBS supplemented with 2 mg of DNase-free RNase at room temperature in darkness. Flow cytometry analyses were performed in the re-suspended cells using a flow cytometer (Central Services, University of Granada, Spain) [41].

### 2.5. Clonogenic Assay

Cells were reseeded in 6-well plates at a density of 3000 cells/well and incubated for 72 h in the absence or presence of increasing BME concentrations (5, 15, 30, and 50 µg/mL) (*n* = 10). Thereafter, colonies were fixed with 2.0% paraformaldehyde, stained with crystal violet (0.5% *w*/*v*), and counted using ImageJ software version 1.54k (National Institutes of Health, Bethesda, MD, USA).

### 2.6. Cell Signaling Assessment

The protein expression of phosphorylated/non-phosphorylated intracellular signaling kinases was assessed following incubation of the chondrocytes in the absence (control) or presence of either sonicated or non-sonicated BMEs (30 µg/mL) for 30 min or 24 h (*n* = 4). Plates were flash-frozen in liquid nitrogen and processed following procedures described previously [42]. Following BME treatment, cells were lysed with radioimmunoprecipitation assay (RIPA) buffer supplemented with phosphatase and protease inhibitors, 10 mM of sodium fluoride, 10 mM of sodium pyrophosphate, 1 mM of sodium orthovanadate, 1 mM of egtazic acid (EGTA), 20 nM of okadaic acid, 10 μg/mL of aprotinin, 10 μg/mL of leupeptin, and 10 μg/mL of pepstatin. Total protein concentration was measured using the bicinchoninic acid method. Proteins (40 μg) were separated by sodium dodecyl sulfate–polyacrylamide gel electrophoresis (SDS-PAGE), transferred onto nitrocellulose membranes, and immunoblotted with antibodies specific to phosphorylated and non-phosphorylated states. GAPDH was used as a protein loading control. Immunoblots were conducted using an enhanced chemiluminescence detection method in a Chemidoc MP Imaging System (Bio-Rad, Madrid, Spain). Quantification was performed through densitometry analysis using the ImageLab v6.0.1 Software (Bio-Rad, Madrid, Spain).

### 2.7. Gene Profiler PCR Array

The expression of genes involved in bone formation and development was assessed following incubation in the presence or absence of BMEs (30 μg/mL, 24 h) (*n* = 4). Total RNA was isolated from chondrocytes using the GeneJET™ RNA Purification Kit (Thermo Scientific, Madrid, Spain). The amount of total RNA was measured using a NanoVue™ Plus Spectrophotometer (GE Healthcare Bio-Sciences, Madrid, Spain) and quality was verified through 1% agarose gel electrophoresis. Genomic DNA was removed using the RapidOut™ DNA Removal Kit (Thermo Scientific). Lastly, 1 μg of total RNA from each sample was reverse-transcribed using Maxima First Strand cDNA Synthesis Kit for RT-qPCR (Thermo Scientific). Expression of bone formation-related genes was assessed using the commercially available ossification and bone remodeling Tier 1 H96 PCR array (BioRad, Hercules, CA, USA) to provide a global view of potential effector targets. Data analysis was conducted through g:Profiler Web Server, and Gene Ontology (GO) enrichment analysis of biological processes on the input gene list was conducted using the g:GOSt functional profiler tool (University of Tartu, Tartu, Estonia) [43].

### 2.8. In Vivo Research and Experimental Design

All experimental procedures involving animal research reported in the present study were carried out in strict adherence to the Spanish regulations (RD 53/2013) (approval codes 23/05/2016/088 and 16/11/2020/132) and in accordance with the European regulation on the protection of animals used for scientific purposes (Directive 2010/63/EU).

Oncins France strain A (OFA) 21-day-old male Sprague–Dawley rats provided by Charles Rivers (Orleans, France) (*n* = 64) were assigned either to non-restricted (ad libitum) feeding (NR, *n* = 8) or a diet-restricted regimen consisting of 70% of the food provided the day before to non-restricted animals after weight correction (RR, *n* = 56). The criteria for food restriction were based on previous research showing the deleterious effects of 70% food intake on axial and appendicular bone quantity and quality outcomes in fast-growing rats [23]. During this three-week period, the RR and NR group animals were fed a standardized commercial diet for growing rodents with sufficient mineral and vitamin contents (AIN93G), had free access to de-ionized water, and were kept under standardized housing conditions (individual housing, 22 °C, 50% relative humidity, 12 h light/dark cycle).

Following the three-week period, the animals from the NR and RR groups (*n* = 8 for each group) were sacrificed through exsanguination under intraperitoneal anesthesia in post-absorptive conditions to confirm the deleterious effects of restricted food intake on bone health and longitudinal growth. To this end, the animals were measured and appendicular long bones (tibias) were collected and stored at −20 °C for ensuing densitometry and micro-CT analyses.

The rest of the food-restricted rodents (*n* = 48) were placed on a two-week refeeding period (short-term catch-up growth). During the refeeding period, previously restricted animals were provided the adapted version of the standardized rodent maintenance diet “AIN93M” [44], alone (CTR, *n* = 20) or supplemented with BMEs (BME, *n* = 20). Both diets had the same caloric and macro- and micronutrient contents. The remaining subset of animals was kept under 70% food intake and received the control diet (stunted controls) (RR, *n* = 8). Upon completion of the two-week refeeding period, eight animals from each refeeding group as well as the eight food-restricted animals were sacrificed and measured following the abovementioned procedures, and tibias were isolated.

**Figure 1:**
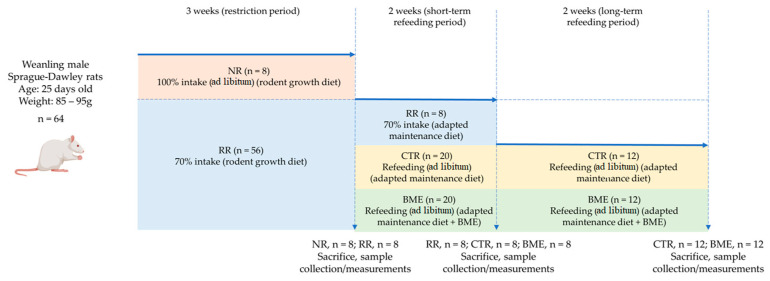
Research model of diet-induced catch-up growth. BME—group refed with the diet supplemented with whey protein concentrate enriched in bovine milk exosomes; CTR—group refed with the control diet; NR—non-restricted group; and RR—restricted group.

Lastly, the refeeding period was extended for another two weeks in the remaining rats (CTR = 12; BME = 12) (long-term catch-up growth). At the end of the four-week refeeding period, the rats were sacrificed, and tibias were removed following the procedures described above. Since the deleterious effects of food restriction on longitudinal growth and bone outcomes were demonstrated following the three-week restriction and two-week refeeding periods, no animal was kept under food restriction during the extended period to ensure compliance with standards of animal care and wellbeing. The day before the sacrifice, body composition (fat mass and lean mass) was measured through magnetic resonance imaging (MRI) using an Echo MRI Body Composition Analyzer system (EchoMRI-700™, Echo Medical Systems, Houston, TX, USA) and values were compared with measurements at the start of the refeeding period to calculate growth velocity rates. A scheme summarizing these experimental procedures is presented in Figure 1.

### 2.9. Ex Vivo Measurements, Densitometry, and Micro-CT Analyses

Bone mineral density (BMD), bone mineral content (BMC), and length of isolated appendicular bones (tibias) as well as the total body length of rats (from the tip of the nose to the end of the second caudal vertebra) were assessed through dual-energy X-ray absorptiometry (DXA) (UltraFocusTM DXA System, Hologic, Tucson, AZ, USA). All of the analyses were conducted by the same technician.

Micro-CT analyses of isolated tibias were performed using a cabinet cone-beam Micro Compute Tomograph µCT 100 (SCANCO Medical AG, Brüttisellen, Switzerland). Tibia metaphysis was scanned at the secondary spongiosa right below the growth plate to evaluate the trabecular bone structure. Growth plate CT scans were segmented based on their grey-scale values and morphometric parameters (thickness, surface, and volume) at the region of interest were computed using the maximum fitted spheres method [45]. Imaging was conducted using a tungsten target at 70 kVp and 11 µA. Scans were performed with a voxel size of 10 µm and an exposure time of 300 ms. To partially suppress noise, acquired data underwent smoothing using a three-dimensional constrained Gaussian filter, employing a finite filter support of one voxel and filter width of *σ* = 0.8. Image segmentation was conducted to differentiate bone from the background using a global thresholding algorithm of 430 mg HA/ccm for the trabecular bone. The analyzed tibia trabecular microarchitecture parameters consisted of bone volume fraction (BV/TV, ratio), bone surface/volume fraction (BS/BV), trabecular thickness (Tb. Th., mm), trabecular space/separation (Tb. Sp., mm), and connectivity density (Conn. D., 1/mm^3^).

### 2.10. Statistical Analysis

The results were expressed as mean and standard deviation (mean ± SD). The normal distribution of continuous variables was explored using the Shapiro–Wilk test. The statistical significance of differences observed across experimental treatments in in vitro tests was explored using one- or two-way ANOVA with multiple comparisons. Follow-up comparisons of means with every other mean or control mean were performed using Tukey’s or Dunnett’s tests. A two-way ANOVA with post hoc Fisher’s LSD test was performed to evaluate changes in body weight throughout experimental periods in the in vivo model. Whenever normality or equal distribution of the variance (Bartlett’s homoscedasticity test) were not achieved, Brown–Forsythe and Welch ANOVA tests were performed instead. Student t-tests or Mann–Whitney U-tests were performed to enable pair-wise comparisons in different groups. *p*-values < 0.05 were considered statistically significant in all analyses, while *p*-values < 0.10 were noted as a trend. All analyses were conducted in GraphPad Prism^TM^ v9.1.2 (GraphPad Software Inc., San Diego, CA, USA).

## 3. Results

**Figure 2:**
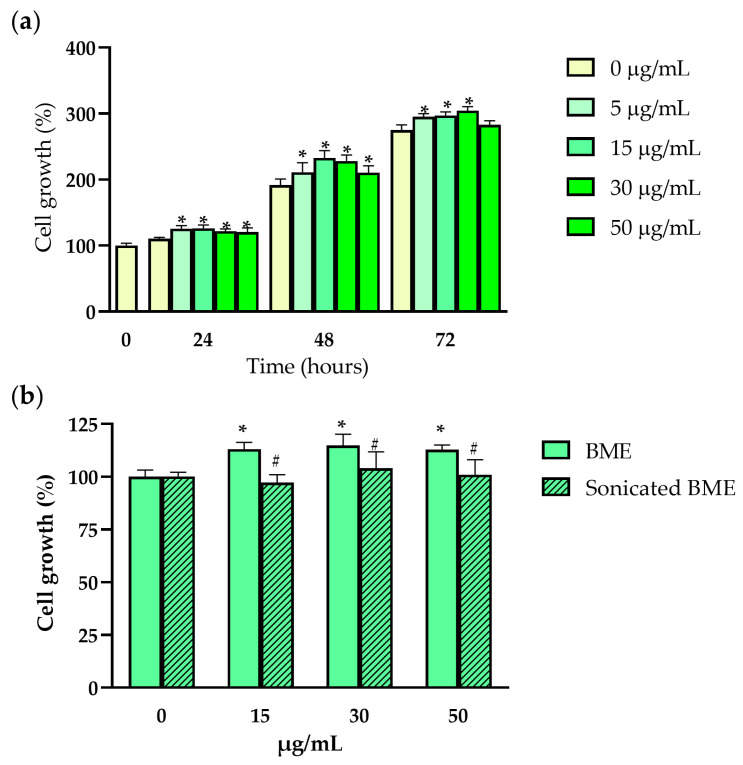
The effects of BMEs on the growth of human chondrocytes. (**a**) Bar plot showing the effects of bovine milk exosomes (0–50 µg/mL; 72 h incubation) on C28/I2 metabolic activity in the MTT test (*n* = 6); (**b**) impact of sonication on bovine milk exosome biological function in a repeated MTT test (0–50 µg/mL; 48 h incubation) (*n* = 4). Data represented as mean ± SD. BME—whey protein concentrate enriched in bovine milk exosomes. * *p*-value < 0.05 compared to the control (0 µg/mL); ^#^ *p*-value < 0.05 compared to non-sonicated bovine milk exosomes.

We tested in vitro the effects of BMEs on human chondrocyte growth. To bypass the low mitotic activity of primary cultures of human chondrocytes, the immortalized cell line C28/I2 was used in all in vitro experiments. C28/I2 chondrocyte growth was evaluated following treatment with increasing doses of BMEs using the MTT test. While no single BME dose negatively impacted cell growth, BME doses of 5–50 µg/mL significantly increased the number of metabolically active cells after 24 and 48 h of incubation, while doses between 5 and 30 µg/mL were significant at 72 h (Figure 2a). To explore whether ultrasonication may blunt BME biological function, cells were incubated for 48 h with increasing concentrations of either ultrasonicated or non-sonicated BMEs. As shown in Figure 2b ultrasonication of BMEs led to null effects on chondrocyte growth in treated cells, while non-sonicated intact BMEs positively impacted the number of metabolically active cells at all tested doses (15, 30, and 50 µg/mL).

**Figure 3:**
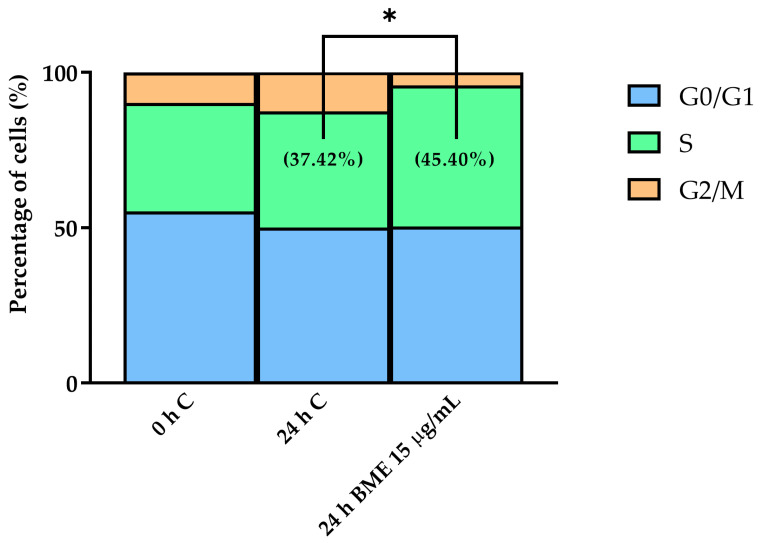
The effects of BMEs on cell cycle profile in human chondrocytes. Control: 0 and 24 h incubation; BME: 15 µg/mL, 24 h incubation. Data represented as mean (*n* = 10). BME—whey protein concentrate enriched in bovine milk exosomes. * *p*-value < 0.05 compared to the control.

To explore how the positive effects of BMEs on cell growth were reflected by changes in cell cycle phases, we incubated chondrocytes in the presence or absence of BMEs and determined the DNA content through propidium iodide staining and flow cytometry analysis. The selected dose consisted of that eliciting the largest effect in the MTT assays (15 µg/mL). At early stages of bone growth and repair, chondrocytes display changes in their cell cycle, promoting the transition from the G1 to S phases to support active protein synthesis [46]. BME treatment increased the number of cells in the synthesis phase (S) compared to the control (24 h C) (Figure 3 and Figure S3). Specifically, the 15 µg/mL BME treatment increased the percentage of cells in the synthesis phase (S) of the cell cycle from 37.42% to 45.40% (Figure 3).

**Figure 4:**
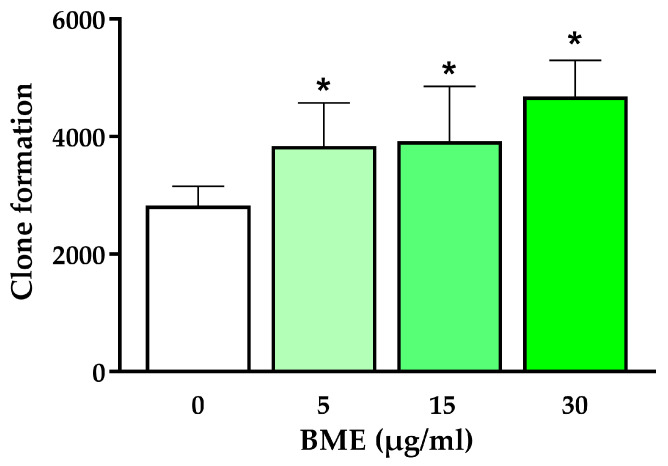
The effects of BMEs on clone formation in human chondrocytes. Data represented as mean ± SD (*n* = 10). BME—whey protein concentrate enriched in bovine milk exosomes. * *p*-value < 0.05 compared to the control.

To better understand BME-mediated effects on chondrocyte proliferation, a clonogenic assay was performed in the absence or presence of BMEs. Doses ranging from 0 to 30 µg/mL were tested, and BME treatment was shown to promote colony formation in the chondrocytes, with 30 µg/mL being the dose eliciting the most pronounced effects (Figure 4).

**Figure 5:**
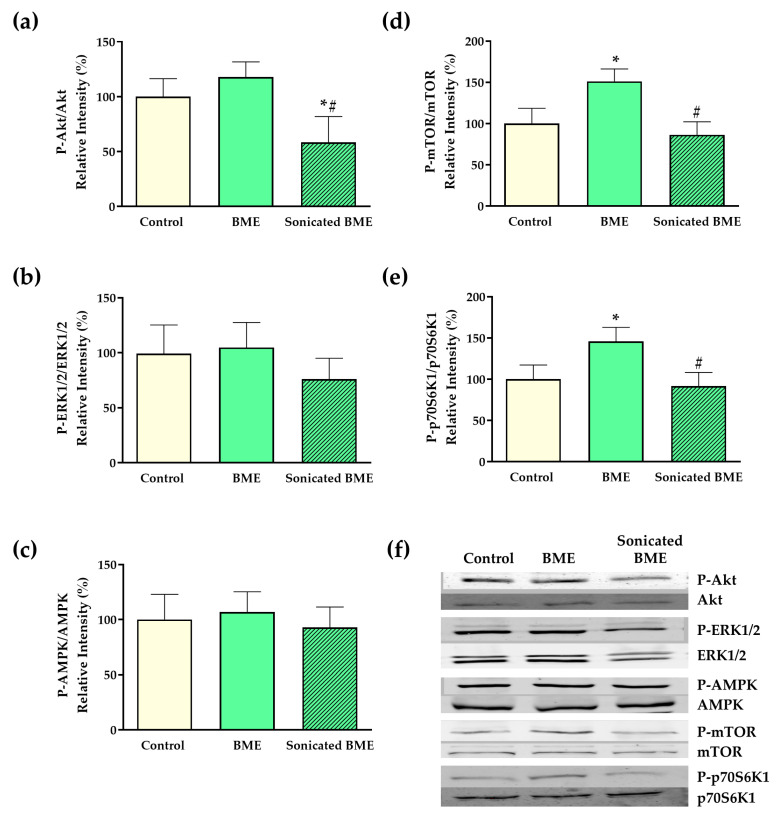
The effects of BMEs on intracellular signaling mechanisms. Bar plots showing the effects of BME and sonicated BME on (**a**) Akt, (**b**) ERK1/2, (**c**) AMPK, (**d**) mTOR, and (**e**) p70S6K; (**f**) representative western blot images of signaling components. Control: 0 µg/mL, 24 h; BME/Sonicated BME: 30 µg/mL, 24 h. Data represented as mean ± SD. BME—whey protein concentrate enriched in bovine milk exosomes. * *p*-value < 0.05 compared to the control; ^#^ *p*-value < 0.05 compared to non-sonicated bovine milk exosomes.

The expression of main phosphorylated and non-phosphorylated signaling proteins involved in intracellular translational machinery was assessed to explore potential mechanisms that might underpin BME biological effects on chondrocyte proliferation and activity. Enhanced Mechanistic Target for Rapamycin (mTOR) expression was shown in BME-treated chondrocytes compared to the control treatment (Figure 5d). Further, these results correlated with the upregulation of a major mTOR downstream substrate target, ribosomal protein s6 kinase-1 (p70S6K1) (Figure 5e), along with a trend (*p* = 0.06) towards increased expression of the mTOR upstream regulator Akt (Figure 5a). On the other hand, no impact was observed on the expression of different upstream effectors (ERK1/2 and AMPK) (Figure 5b,c). To further consolidate the role of the mTOR-Akt pathway in BME-mediated effects, mTOR and PI3K-Akt inhibitors rapamycin and LY29402 were used in replicated MTT tests. As a result, both rapamycin and LY29402 were shown to blunt BME-mediated effects on chondrocyte growth (Figure S4).

**Figure 6:**
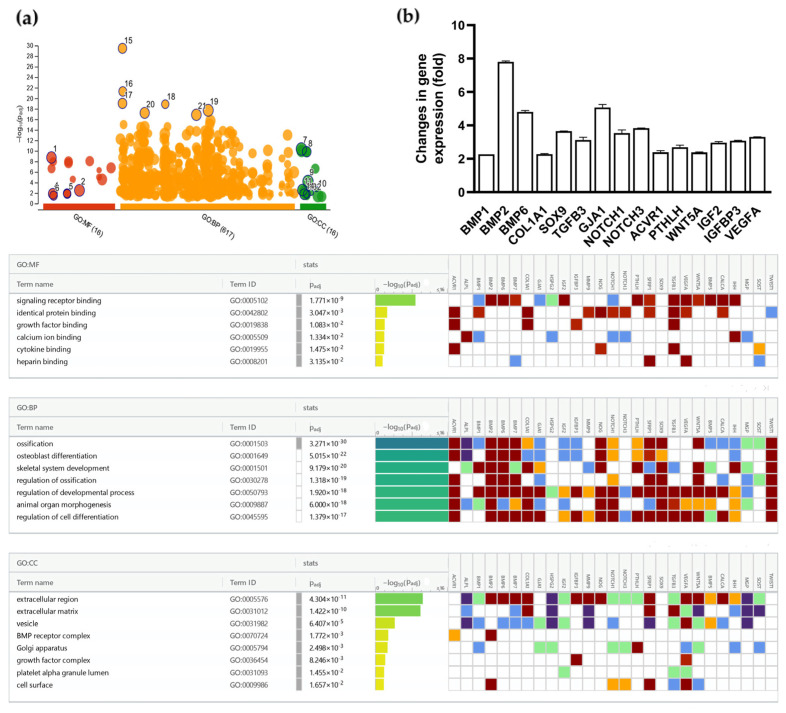
The effects of BME incubation (30 µg/mL, 24 h) on the expression of genes involved in bone formation and development. (**a**) Gene Ontology (GO) functional enrichment analysis results; (**b**) genes at least 2-fold-overexpressed following BME incubation. Color codes gene ontology evidence can be consulted in reference [43]. Data represented as mean ± SD. BME—whey protein concentrate enriched in bovine milk exosomes.

Lastly, a gene profiler PCR array revealed promoted expression of several genes involved in bone formation and development in BME-treated chondrocytes following 24 h of incubation. Particularly, we observed promoted expression of SOX9 and BMP-2, two genes demonstrated to play crucial roles in chondrocyte proliferation and maturation during endochondral ossification [47,48] (≈3.5- and 9-fold increase, respectively). Overexpressed genes and GO functional enrichment analysis results are presented in Figure 6. Overall, differentially expressed genes (DEGs) were mainly involved in functional pathways linked to ossification, osteoblast differentiation, and skeletal system development.

In light of results showing improved chondrocyte proliferation and potential anabolic mechanisms involved, we set out to evaluate how these findings might apply to support bone health and linear growth in a model of diet-induced catch-up growth in stunted rats. Sprague–Dawley rats (*n* = 64) were fed ad libitum (NR, *n* = 8) or were placed on dietary restriction for three weeks (70% intake compared to non-restricted rats) (RR, *n* = 56). Long bone length is the best indicator of stature in humans [49], and lower extremity long bone lengthening assessment is instrumental in studying infant growth rates [50]. Hence, tibias were selected as representative appendicular long bones in all analyses. At the end of the restriction period, RR animals displayed ≈44% lower mean body weight (BW) and ≈14% decreased mean length compared to the NR group (Figure S5). As a consequence of the diet restriction, significant length decrements were observed in tibias (Figure S6). In the same manner, densitometry analyses revealed significantly lower BMD and BMC values in assessed tibias of food-restricted animals (Figure S6). Micro-CT analyses revealed that the three-week food restriction period led to higher tibia trabecular thickness and trabecular space/separation, along with decreased trabecular number and connective density in diet-restricted rats (Figure S7). Lower trabecular number and connective density paired with increased trabecular separation are indicative of poor bone trabecular structure in these animals and have been previously reported following restricted food intake during growth [18,23]. Lastly, significant decrements were evident in all growth plate micro-CT parameters assessed in restricted compared to non-restricted rats (Figure S8).

Once we evaluated our animal models, two groups of previously restricted animals underwent a 2-week refeeding period to study whether the positive effects of BMEs on chondrocyte proliferation may translate to improved growth plate activity during the active growth period. During this time period, they were assigned to receive either a BME-supplemented diet (experimental group, BME) or the non-supplemented control diet (control group, CTR) with the same caloric content and macro/micronutrient composition, while another group was kept under food restriction receiving the control diet (70% intake), thus acting as the stunted control group. A two-week refeeding period was chosen to achieve sufficiently incomplete closure of the physis, thus enabling clear visualization of changes in the growth plate during the active growth period.

Throughout the refeeding period, both refeeding groups regained weight to a similar extent (Figure S9c) and no difference in the amount of food consumed or feed efficiency was observed between them (Figure S9a,b). Upon completion of the two-week refeeding period, eight animals from each group were sacrificed, and tibias were collected for analysis. As revealed in DXA analyses, total body length and isolated tibia length were significantly higher in animals from both refeeding groups than those observed in restricted animals (RR) (Table 1), while no difference was observed between refeeding groups. Similarly, improved tibia BMC and BMD were observed in both refeeding groups compared to the RR rats. Notably, a significant increment (≈3.8%) in tibia BMD was observed in the BME-supplemented animals compared to the control refeeding group (Table 1).

**Figure 7:**
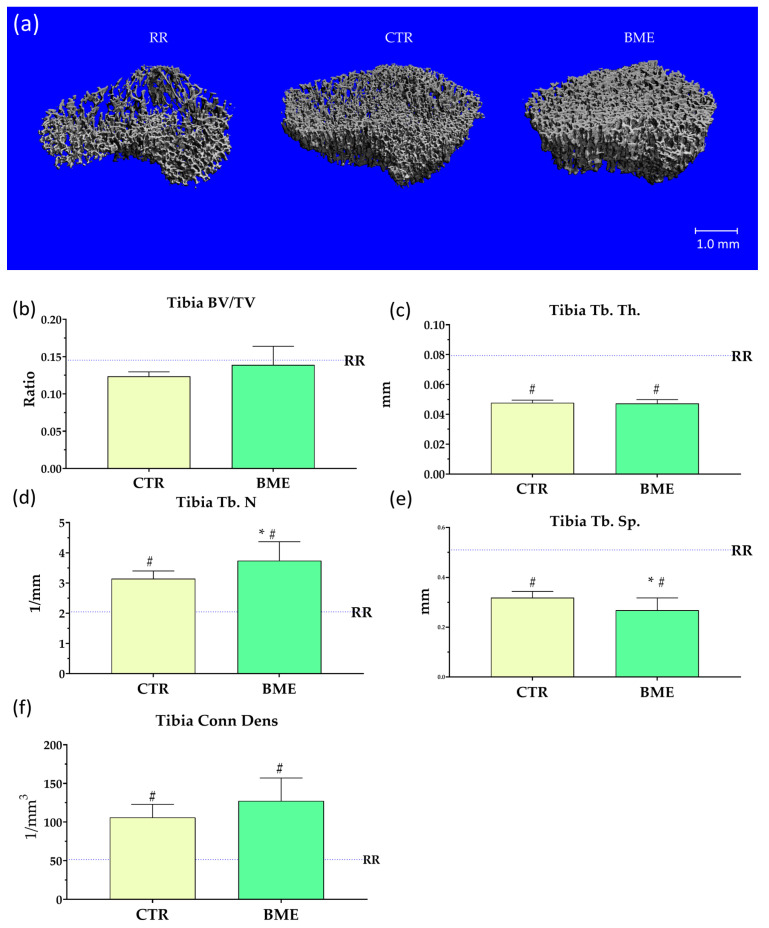
Representative images (**a**) and micro-CT parameters (**b**–**f**) of tibia trabecular structure upon completion of the two-week refeeding period. Data represented as mean ± SD. BME—group refed with the diet supplemented with whey protein concentrate enriched in bovine milk exosomes; BV/TV—bone volume to total volume ratio; Conn. Dens.—connective density; CTR—group refed with the control diet; RR—restricted group; Tb. N.—trabecular number; Tb. Sp.—trabecular separation; and Tb. Th.—trabecular thickness. * *p*-value < 0.05 compared to the control refeeding group (CTR); ^#^ *p*-value < 0.05 compared to the restricted group (RR).

As observed in micro-CT scans of tibia secondary spongiosa (Figure 7a), BME-fed rats exhibited a significantly higher number of trabeculae per unit of length (≈16%) (Figure 7d) along with significantly decreased trabecular separation (≈16%) (Figure 7e) compared to rats refed with the control diet. While ≈20% increased connective density (Figure 7f), and ≈12% increased BV/TV ratio (Figure 7b) were observed in BME-supplemented rats, no statically significant difference between refeeding groups was found. Compared to restricted animals (RR), significantly higher connective density and trabecular number (Figure 7d,f) along with notably lower trabecular thickness and separation (Figure 7c,e) were evident in both ad libitum refed groups.

**Figure 8:**
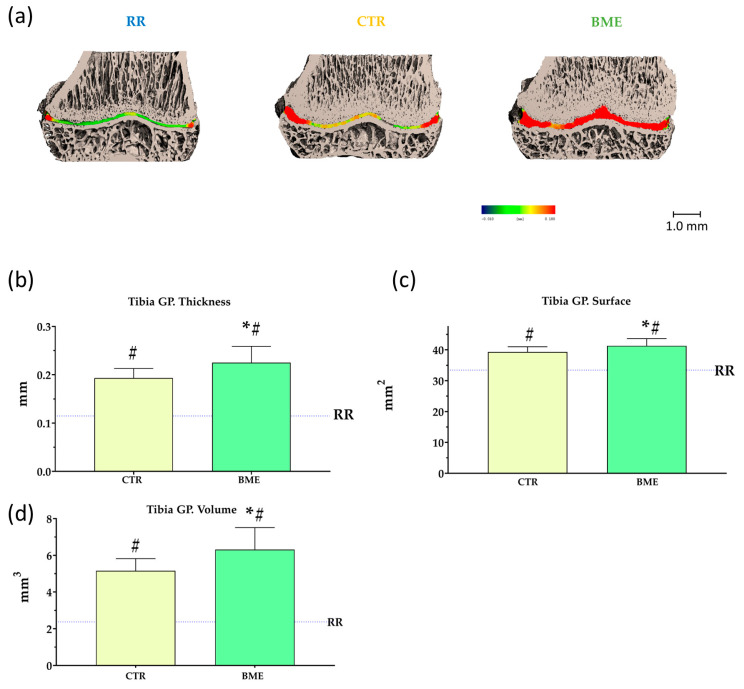
Representative scans (**a**) and micro-CT parameters (**b**–**d**) of the tibia growth plate upon completion of the two-week refeeding period. Data represented as mean ± SD. BME—group refed with the diet supplemented with whey protein concentrate enriched in bovine milk exosomes; CTR—group refed with the control diet; GP—growth plate; and RR—restricted group. * *p*-value < 0.05 compared to the control refeeding group (CTR); ^#^ *p*-value < 0.05 compared to the restricted group (RR).

Micro-CT results of the tibia growth plates are presented in Figure 8. Reintroducing sufficient food provisions notably improved growth plate micro-CT parameters, which were previously impaired by food restriction (shown in Figure S7). On the other hand, poorer growth plate thickness, surface, and volume were clearly observed in rats placed on 5 weeks of food restriction compared to ad libitum refed animals (Figure 8b–d). Notably, significantly higher growth plate thickness (≈16%), surface (≈5%), and volume (≈22%) were observed in BME-supplemented rats compared to those refed with the control diet (Figure 8b–d).

**Figure 9:**
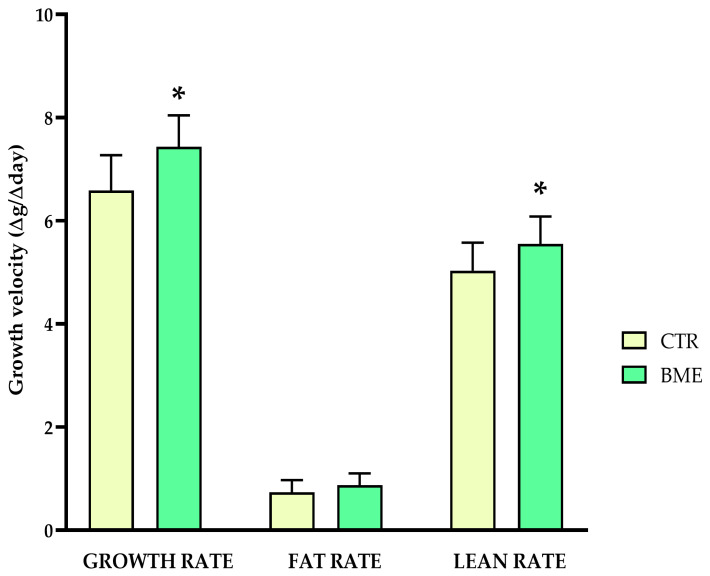
Growth velocity of the experimental and control animals. Data represented as mean ± SD. BME—group refed with the diet supplemented with whey protein concentrate enriched in bovine milk exosomes; CTR—group refed with the control diet. * *p*-value < 0.05 compared to the control refeeding group (CTR).

Whether higher growth plate activity might have positive repercussions on the attained bone length of rats following a more prolonged period of epiphyseal maturation was tested in an ensuing long-term experiment. In a similar fashion, the remaining animals were placed on ad libitum feeding with either the adapted diet supplemented with BMEs (BME, *n* = 12) or the control diet (CTR, *n* = 12). At the end of the four-week refeeding period, significantly higher growth velocity, defined as weight change per day measured on the first refeeding day and the day before sacrifice, paired with an increase in lean mass growth rate, were observed in BME-supplemented rats compared to animals fed the control diet (Figure 9).

**Figure 10:**
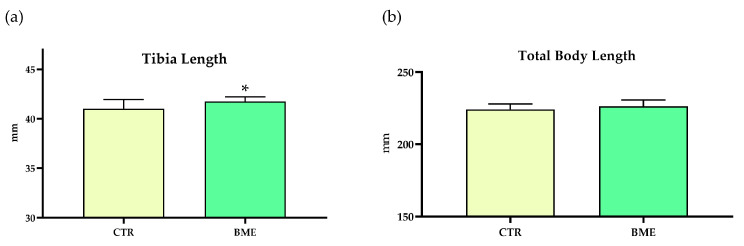
The effects of the four-week refeeding period on total body (**b**) and tibia length (**a**). Data represented as mean ± SD. BME—group refed with the diet supplemented with whey protein concentrate enriched in bovine milk exosomes; CTR—group refed with the control diet. * *p*-value < 0.05 compared to the control refeeding group (CTR).

Tibia bones were removed, and densitometry and micro-CT analyses were conducted at the end of the four-week refeeding period. Notably, tibias of BME-supplemented animals were significantly longer (≈2%) than those of rats assigned to the control refeeding diet (Figure 10a), yet no change in total body length was observed between refeeding groups (Figure 10b). Further, improvements in tibia length were apparent without compromising tibia bone quality or quality, as denoted by densitometry and micro-CT analyses (Table S2).

## 4. Discussion

Linear growth is a key determinant of child development, and severe undernutrition is known to result in longitudinal growth stagnation or stunting. Once food provisions are restored, a process of accelerated growth (catch-up growth) occurs; however, the characteristics of the nutrition provided are key to supporting healthy growth phenotypes. In the present research, we provided the first evidence supporting the functional role of a whey protein concentrate enriched in bovine milk exosomes (BMEs) in promoting proliferation in C28/I2 human chondrocytes. Further, we tested the potential effects of BMEs on longitudinal growth in a validated model of diet-induced catch-up growth in stunted rats, observing positive effects of two-week BME supplementation on tibia bone mineral density (BMD) and microarchitecture parameters, namely trabecular number (Tb. N.) and separation (Tb. Sp.), compared to rats fed the non-supplemented control diet. Notably, significant improvements in growth plate thickness, surface, and volume were apparent in BME-supplemented compared to non-supplemented rats. Extending the refeeding period with the BME-supplemented diet for another two weeks was shown to deliver improvements in tibia length paired with higher growth velocity and higher lean mass growth rates compared to non-supplemented animals without compromising bone densitometry and micro-CT parameters, which overall denotes positive effects of BME supplementation on supporting bone health and development during catch-up growth.

There remain many open questions pertaining to how different dietary components modulate longitudinal growth [51]. Differences in cortical and trabecular bone microstructure and tibia growth plate height were observed in undernutrition-induced stunted rats (60% intake for 36 days) fed whey or casein protein for 24 or 40 days during catch-up growth, with whey protein intake suggested to help circumvent long-term complications associated with accelerated growth patterns [21]. Specifically, whey protein intake was observed to maintain a higher epiphyseal growth plate for a longer time; however, casein intake promoted better bone microstructural properties during the short-term (24-day) experiment [21]. In the same vein, better maintenance of the epiphyseal growth plate was reported in stunted rats (60% intake for 10 days) assigned to whey versus soy protein (14 days) during catch-up growth [52]. Partially replacing sn-1,3 palmitic acid with β-palmitate (esterified in position sn-2) in diets provided to undernutrition-induced stunted rats (60% intake for 17 days) during refeeding (nine days) was shown to increase humerus length, and tended to increase growth plate height with significant positive effects on some cortical and trabecular bone parameters [22]. In a similar fashion, recent research supports the beneficial effects of slow digestible carbohydrates on bone mass accrual and architecture compared to fast digestible carbohydrate intake (four weeks) in an animal model of undernutrition-induced stunting (70% intake for four weeks) during catch-up growth [23]. However, it may be challenging to directly compare our in vivo results with those observed in cited previous studies exploring how modulating dietary components differentially impact growth and bone quality during catch-up. Specifically, different bones analyzed (i.e., humerus vs. tibia), restriction protocols (i.e., 60% vs. 70% food intake), and refeeding periods differentially impact bone growth and quantity/quality measurements in rat experiments. Further, the timing when measurements are performed is key to identifying potential changes in growth plate activity, and bone quality and length parameters.

Research on the modulation of macronutrient composition of diets is oftentimes driven by a growing body of evidence supporting the positive effects of milk and dairy product intake on linear growth in children and the interest in elucidating mechanisms involved [53]. However, not only macronutrients but also different components might contribute to these effects, with bovine milk exosomes being particularly attractive due to their bioavailability and unique cargos and composition that might convey beneficial biological effects at multiple levels [25]. Nonetheless, no study to date has assessed bovine milk exosome effects on chondrocyte proliferation, or bone elongation and development during catch-up growth.

We observed that incubation with BMEs was associated with increased cell growth and clone formation, which was also reflected by the higher number of cells in the s-phase in immortalized human C28/I2 chondrocytes, which may be driven by mTOR-Akt signaling. The effects on cell growth and translational signaling were blunted with ultrasonication of exosomes, thus further supporting the functional role of intact bovine milk exosomes in promoting chondrocyte proliferation. On this point, ultrasonication is a process known to disrupt exosomal membranes leading to compromised uptake mechanisms in cell cultures and depletion of different cargos with functional properties (i.e., miRNAs) [54]. Positive effects on chondrocyte proliferation may be particularly important during active growth periods since the rate of chondrocyte proliferation is considered the primary factor contributing to longitudinal bone growth [37]. Chondrocytes initially divide to promote cartilage tissue expansion to eventually cease proliferating and differentiate into hypertrophic chondrocytes, which drive cartilage matrix mineralization during endochondral ossification [13]. Both chondrocyte proliferation and differentiation processes are partially governed by mTOR signaling, as supported by experiments observing impaired proliferation and hypertrophy, either with rapamycin administration (mTOR inhibitor) or leucine (mTOR promotor) restriction in mouse chondrogenic cells (ATDC5) [55,56]. In fact, the administration of rapamycin has been shown to markedly retard growth and alter growth plate parameters in rats [57,58]. Additionally, enhanced expression of several genes involved in pathways related to bone formation and development was observed in the gene profiler PCR array and Gene Ontology (GO) functional enrichment analysis in BME-treated human chondrocytes. Altogether, our in vitro observations support the role of BMEs in promoting human chondrocyte proliferative properties through mTOR signaling and potential effector targets, which might contribute to cartilage tissue expansion during longitudinal bone growth.

The promising findings observed in cell culture experiments were tested in a model of diet-induced catch-up growth in stunted rats. We validated our research model documenting profound deleterious effects of three-week reduced food intake (70% intake) on the tibia growth plate and tibia densitometry and micro-CT parameters. Lower growth plate thickness, volume, and surface documented in the present research are consistent with delayed growth plate senescence observed in different studies conducted in food-restricted growing rodents [53,59,60]. Regarding bone health parameters, decreased tibia mineral content and density (BMC and BMD) have been reported in previous research exploring similar [23] or longer duration [61] food restriction protocols, yet mixed results are available in the literature [18]. Decrements in long bone trabecular number (Tb. N.) and increased trabecular space/separation (Tb. Sp.) have been consistently reported as features of impaired trabecular bone microstructure in previous studies conducted in growing rats or mice subjected to food restriction protocols [18,21,62]. Extending the restriction period to five weeks further delayed growth plate senescence and compromised densitometry and trabecular parameters compared to refed animals, thus further validating our research model of diet-induced stunting throughout childhood and adolescence, the most active growth periods.

Upon completion of the two-week refeeding period, rats averaged ≈ 60 days of age. This time frame is typically regarded as late adolescence in rodents and represents a pivotal moment when body growth begins to decelerate [63]. Two-week refeeding with either the BME-supplemented or the control diet substantially recovered body and tibia growth patterns compared to animals kept under restriction. Refed animals also presented higher tibia BMC and BMD, as well as evident improvements in trabecular microstructure (Figure 7) denoted by the higher number of trabeculae (Tb. N.), lower trabecular separation (Tb. Sp.), and increased connective density (Conn. Dens). Lower trabecular thickness (Tb. Th.) and unchanged bone volume to total volume ratio (BV/TV) in tibias are divergent from previous reports in long bones [21], yet these might be conceived as a compensatory mechanism aimed at maintaining tibia bone structure under nutritional stress. Notably, BME supplementation was linked to higher tibia BMD and, particularly, improved Tb. N. and Tb. Sp. compared to refeeding with the control diet. In light of previous research reporting impaired bone quality during short-term catch-up growth [18], which may be dependent on the composition of the diet provided [21,52], BME supplementation might be beneficial for diets aimed to recover growth patterns without compromising bone health during active growth periods.

We observed differential effects of the experimental and control diets on tibia growth plate micro-CT parameters upon completion of the two-week refeeding period. While both refeeding groups presented increased growth plate thickness, surface, and volume compared to animals kept under restriction, values were significantly higher in rats fed with the BME-supplemented diet compared to the control diet. Both refeeding groups displayed similar total body and tibia length, yet differences in growth plate parameters indicated a more active (younger) growth plate in BME-supplemented rats, suggesting better growth potential [37]. In our view, the most remarkable finding of the present research consists of the significant increment in tibia length of BME-supplemented animals compared to those assigned to receiving the control diet when the refeeding period was extended for another two weeks, albeit differences in total body length were not statistically significant. While long bone length is the most important determinant of attained height in humans [49], other bones contribute to body length measured from nose to tail in growing rats, which may contribute to the lack of significant effect observed. This increment in bone length was not produced at the expense of bone quality or mineral density/content as shown in micro-CT and DXA analyses documenting similar values between refeeding groups. Unlike humans, rats’ long bones do not undergo complete epiphyseal closure [63]; nonetheless, rats were ≈80 days old at the end of the extended refeeding period, which is typically considered “young adulthood” in rodents, and correlates to the age when epiphyseal closure is completed in humans [64]. Further, while growth velocity was higher in BME-supplemented rats, this correlated with changes in lean body mass not being accompanied by excess fat gain, which is supportive of a healthier catch-up growth phenotype in these animals. Altogether, these findings indicate that increasing bovine milk exosome content in diets might have significant positive effects on longitudinal bone growth in settings of previous growth restriction.

Mechanisms underlying different effects of dietary components on catch-up growth are typically proposed to involve enhanced calcium absorption [22], improved control of the insulin–glycemic response [23], the impact of different amino acid profiles on bone turnover [21], and other indirect effects mediated through modulation of the gut microbiota [21,22,23]. Bovine milk exosomes are bioavailable following oral intake and have been documented to reach distant body locations [65,66]. In fact, bone has been reported among the main tissues where bovine milk exosomes accumulate following oral gavage in rodents [65]. Thus, both local and systemic effects of bovine milk exosomes on bone development may be conceived.

Direct effects of bovine milk exosomes on bone formation during the active growth period might be linked to their miRNA content. For instance, miRNAs that are highly expressed in bovine milk exosomes [67,68], such as miRNA-29B, miRNA-148a, and miRNA-21, are thought to mediate processes involved in osteogenic differentiation [69,70,71]. Osteoclast-derived exosomal let-7a-5p, a miRNA highly expressed in bovine milk exosomes [67], has been shown to induce hypertrophy in ATDC5 murine chondrocytes [72]. In a similar fashion, miRNA let-7b-5p, another let-7 family member present in bovine milk exosomes [67], has been suggested to promote proliferation in rat chondrocytes [73]. However, further research is needed to elucidate how bovine milk exosome miRNA networks might contribute to the development of chondrocytes and different bone cells.

Of note, bovine milk exosomes might not only help protect and deliver miRNA but also other cargos with potential biological functions, such as proteins [74]. For instance, lactadherin is among the most frequently reported bovine milk exosome protein cargos and has been shown to promote osteogenic differentiation [74] and influence chondrocyte homeostasis [75]. Transforming growth factor *β* (TGF-*β*) is an immunomodulatory protein contained in bovine milk exosomes [76], which has been implicated in epiphyseal growth plate chondrocyte proliferation and maturation processes [77]. How other bovine milk exosome proteins and different components and cargos (i.e., lipids) might contribute to bovine milk exosome biological effects is still an uncharted field of research.

Lastly, the potential indirect effects of bovine milk exosomes on bone development through systemic regulation remain speculative. Bovine milk exosomes might contribute to the maintenance of the gut barrier, thus facilitating better absorption of nutrients key to bone development such as calcium and vitamin D [78]. In alignment, a growing body of evidence supports the role of bovine milk exosomes in modulating the composition of the gut microbiota [79,80,81]. A recent report suggested that oral administration of bovine milk exosomes might upregulate the abundance of certain bacteria involved in mucus remodeling and short-chain fatty acid (SCFA) production such as *Akkermansia muciniphila*, which may be linked to improved maintenance of the gut barrier, decreased cecum pH, and, in turn, promoted mineral and vitamin absorption [79]. Time-course analyses showing liver accumulation of bovine milk exosomes following oral intake [66] might support potential indirect effects on growth through hormonal regulation (i.e., IGF-1) [29]. Overall, interorgan crosstalk might partially mediate mechanisms underlying the herein-reported positive effects of BMEs on longitudinal growth [25]. However, due to the lack of supporting evidence, this remains a hypothesis to be tested in future research.

## 5. Conclusions

Over a two-week period, feeding with a diet supplemented with whey protein concentrate enriched in bovine milk exosomes (BMEs) resulted in significant improvements in growth plate volume, surface area, and thickness as well as positive effects on bone densitometry and microarchitecture parameters in previously food-restricted stunted rats during catch-up growth. Observed benefits in terms of a more active growth plate were shown to translate to higher tibia length when the dietary intervention was extended for another two weeks. Improvements in bone longitudinal growth might be dependent on the effects of BMEs on promoting chondrocyte proliferative properties through mTOR-Akt signaling, as supported by experiments conducted in human chondrocytes. Altogether, our findings highlight the potential of BMEs as a promising dietary component in diets aimed to facilitate the recovery of linear growth patterns without inducing unhealthy growth phenotypes, which may be relevant to children with special nutritional requirements and growth disorders.

## Figures and Tables

**Table 1:** The effects of the two-week refeeding period on body length and tibia densitometry parameters.

Measurements	Animal Group		
	RR	CTR	BME
Total Body Length (mm)	188.8 ± 8.78	208.4 ± 3.52 ^#^	207.8 ± 5.22 ^#^
Tibia Length (mm)	36.37 ± 1.26	38.64 ± 0.66 ^#^	38.77 ± 0.59 ^#^
BMD (mg/cm^2^)	124 ± 6.16	138 ± 3.39 ^#^	143.4 ± 5.24 ^#,^*
BMC (g)	0.192 ± 0.02	0.261 ± 0.007 ^#^	0.271 ± 0.02 ^#^

Data represented as mean ± SD. BMC—bone mineral content; BMD—bone mineral density; BME— group refed with the diet supplemented with whey protein concentrate enriched in bovine milk exosomes; CTR—group refed with the control diet; and RR—restricted group. * *p*-value < 0.05 compared to the control refeeding group (CTR); ^#^ *p*-value < 0.05 compared to the restricted group (RR).

## Data Availability

The data herein presented are available upon reasonable request to the corresponding author.

## Supplementary Materials

The following supporting information can be downloaded at https://www.mdpi.com/article/10.3390/nu16223814/s1, Table S1: Composition of the adapted maintenance rodent diet, Table S2: Tibia densitometry and micro-CT parameters upon completion of the four-week refeeding period, Figure S1: Characterization of the whey protein concentrate enriched in bovine milk exosome, Figure S2: Analysis of bovine milk exosome content in the experimental and control diets, Figure S3: Histograms showing the effects of BMEs on cell cycle profile in human chondrocytes, Figure S4: The effect of BMEs on the growth of human chondrocytes under inhibition of mTOR (Rapamycin) and Akt (LY294002), Figure S5: The effects of three-week 70% dietary intake on body weight and length, Figure S6: The effects of three-week 70% dietary intake on tibia length and densitometry parameters, Figure S7: The effect of three-week 70% dietary intake on tibia trabecular microstructure, Figure S8: The effect of three-week 70% dietary intake on tibia growth plate, and Figure S9: Food consumption and body weight gain during the two-week refeeding period.
