# Supplementary material for: Supplementation with a Whey Protein Concentrate Enriched in Bovine Milk Exosomes Improves Longitudinal Growth and Supports Bone Health During Catch-Up Growth in Rats
Source: Nutrients. 2024 Nov 7;16(22):3814. doi: 10.3390/nu16223814 (PMC11597726; doi:10.3390/nu16223814)
Supplement: Supplementary file 1 [file nutrients-16-03814-s001.zip › nutrients-3270427-supplementary.pdf]

Supplementary 1

ExoView Methods

ExoFlex™ chips (Unchained labs, Pleasanton, CA) specifically capture exosomes via a microarray silicon chip functionalized with antibodies. ExoFlex™ technology permits the addition of custom antibodies via antibody-linker conjugation to peptide-linkers bound to the surface of the chip. Chips were arrayed with capture antibodies (in triplicate) against mouse IgG (isotype negative control) and CD9-Flex 1. To facilitate binding of linker-conjugated antibodies to the chips, linker-conjugated antibodies were diluted 1:100 in solution A (Unchained labs, Pleasanton, CA) at room temperature. A total of 40 µL of diluted antibody-linker conjugate was incubated on each chip surface for 30 minutes at room temperature. Chips were washed following manufacturer recommendations with kit solutions to remove unbound material and prepare for sample incubation. Briefly, 1 mL of solution A (Unchained labs, Pleasanton, CA) was added to each well, and 750 µL of solution within the wells was then discarded. Thereafter, 750 µL of solution B (Unchained labs, Pleasanton, CA) was added to each well, and 750 µL of solution within the wells was then discarded to be replaced with 750 µL of distilled water. Chips were carefully removed and placed in Petri dishes (10 cm diameter) containing distilled water. Chips were washed, dried, and then imaged using a ExoView R200™ reader with the ExoView Scanner™ v3.2.1 software (Unchain labs, Pleasanton, CA). Commercial cheese whey and the whey protein concentrate enriched in bovine milk exosomes (both at 6% solid content) were diluted 1:300 while experimental and control diets were diluted 1:10 in incubation solution (Unchained labs, Pleasanton, CA). Thereafter, 50 µL of diluted sample was applied to each chip, and the plate was sealed and incubated for 16 hours at room temperature in the dark. Chips were then washed three times with solution A. Each of the washing steps was performed in the presence of shaking (500 rpm) for 3 min. Following the last wash, 250 µL of fluorescence-labeled anti-CD9 antibody diluted 1:500 with kit blocking solution (Unchained labs, Pleasanton, CA) were added to each well. The plates were incubated for 1 h at room temperature in the dark. Wells were then washed five times sequentially: the first wash in solution A, the next three washes in solution B, and a final wash in distilled water. Chips were carefully removed and placed in petri dishes (10 cm diameter) containing distilled water. Chips were then dried and imaged using the ExoView R200™ reader using ExoView Scanner™ 3.2.1 software for post-scan. Data were exported using ExoView Analyzer™ v3.2 with fluorescence gating based on control mouse IgG capture.

Table S1 Composition of the adapted maintenance rodent diet

Energy and macronutrients	Adapted maintenance rodent diet
**Energy** (kcal/100 g diet)	402
**CHO** (g/100 g diet)	66.1
• Sucrose (%)	6.8
• Maltodextrins (%)	80.2
• FOS (%)	5.7
**Proteins** (g/100 g diet)	13.2
• Milk protein (%)	27.4
• Soy protein (%)	19.5
• Calcium caseinate (%)	51.4
**Fat** (g/100 g diet)	10.3

• Canola oil (%)	14.5
• Soy oil (%)	42.5
• Medium-chain triglycerides (%)	7.9
• High oleic sunflower oil (%)	29.8

CHO, carbohydrates; FOS, fructooligosaccharides. Remaining percentages include vitamin/mineral premix and inherent components. Adapted to resemble the typical composition of diets supplemented to meet the needs of stunted children (humanized diet).

Table S2. Tibia densitometry and micro-CT parameters upon completion of the four-week refeeding period

	Animal Groups	
**Densitometry Parameters**	**CTR**	**BME**
BMD (mg/cm^2)	156.1 ±4.67	153.7 ±9.59
BMC (g)	0.32 ±0.02	0.32 ±0.01
**Micro-CT Parameters**		
BV/TV (ratio)	0.13 ±0.01	0.13 ±0.02
Tb. Th. (mm)	0.05 ±0.003	0.05 ±0.003
Tb. N. (1/mm)	3.11 ±0.4	3.11 ±0.74
Tb. Sp. (mm)	0.31 ±0.03	0.34 ±0.09
Conn. Den. (1/mm^3)	97.27 ±7.36	96.47 ±18.15

Data represented as mean ± SD. BMC, bone mineral content; BMD, bone mineral density; BME, group refed with the diet supplemented with the whey protein concentrate enriched in bovine milk exosomes; BV/TV, bone volume to total volume ratio; Conn. Dens., connective density; CTR, group refed with the control diet; RR, restricted group; Tb. N., trabecular number; Tb.Sp., trabecular separation; Tb.Th., trabecular thickness.

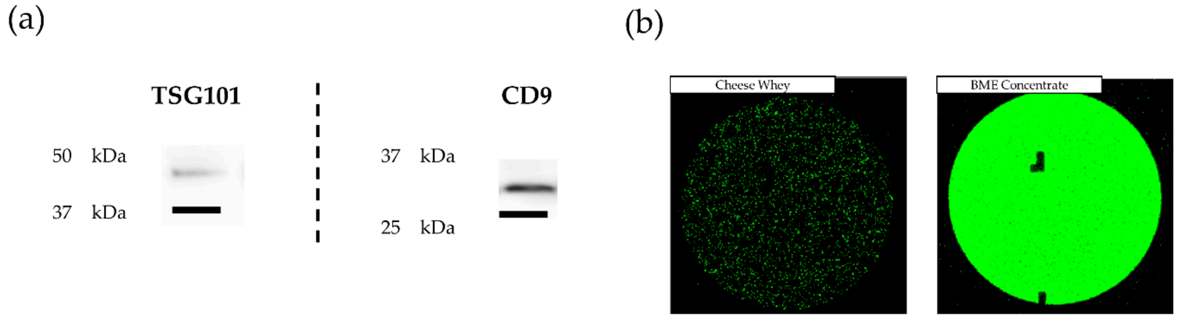

Figure S1. Characterization of the whey protein concentrate enriched in bovine milk exosomes. a) Western blot analysis of exosome markers TSG101 and CD9 in the whey protein concentrate enriched in bovine milk exosomes (BME); b) ExoView analysis of commercial cheese whey and the BME.

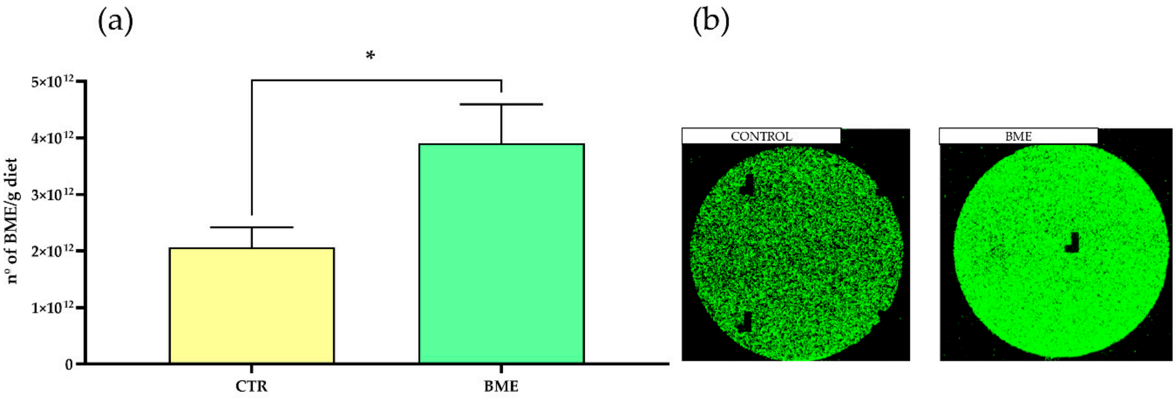

Figure S2. Analysis of bovine milk exosome content in the experimental and control diet. a) Graphical representation and b) ExoView imaging of bovine milk exosome content in experimental and control diets. Data represented as mean ± SD. BME, diet supplemented with whey protein concentrate enriched in bovine milk exosomes; CTR, control diet. * P-value <0.001 compared to control.

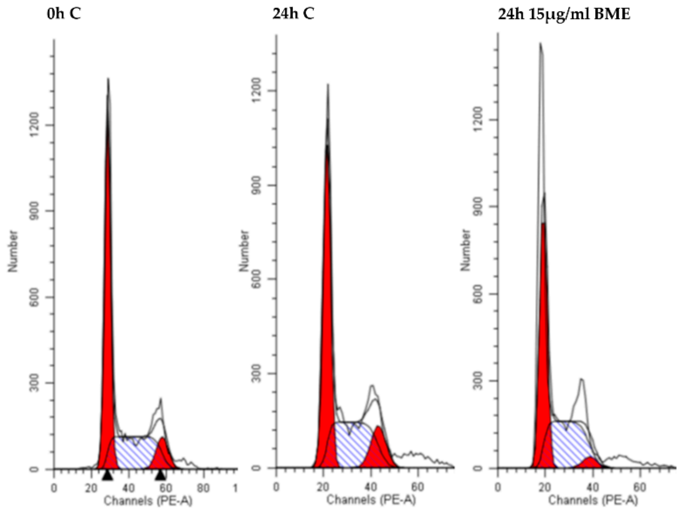

Figure S3. Histograms showing the effects of BME on cell cycle profile in human chondrocytes. Control: 0 and 24 h incubation; BME: 15 µg/mL, 24 h incubation. BME, whey protein concentrate enriched in bovine milk exosomes.

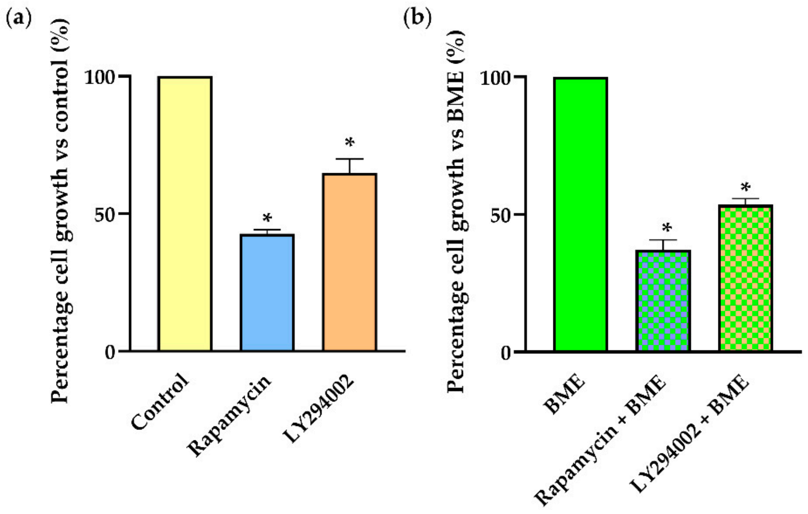

Figure S4. Effects of BME on growth of human chondrocytes under inhibition of mTOR (Rapamycin) and PI3K-Akt (LY294002). a) Rapamycin and LY29002 compromise cell growth through inhibition of mTOR-Akt pathway; b) The positive effects of BME on cell growth are blunted in presence of rapamycin and LY294002. Control: 24 h incubation in absence of BME; BME: 30 μg/mL, 24 h incubation. Data represented as mean ± SD. BME, whey protein concentrate enriched in bovine milk exosomes. * P-value <0.05 compared to control/BME treatment.

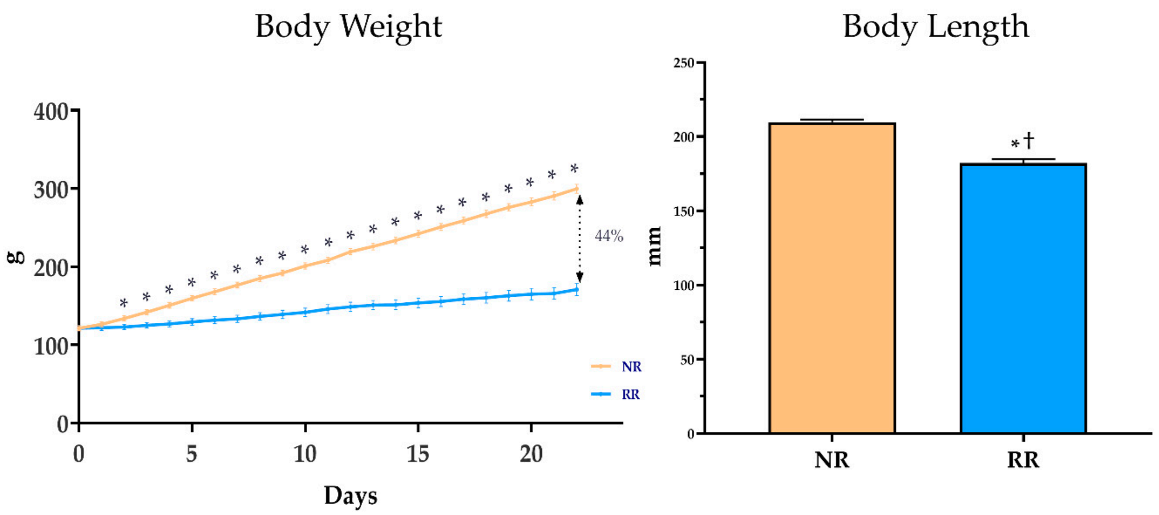

Figure S5. Effect of three-week 70% dietary intake on body weight and length. NR, non-restricted group; RR, restricted group. Data represented as mean ± SD. * P-value <0.05 2-way ANOVA, Fisher's LSD post hoc; † P-value <0.05 Student's t-test.

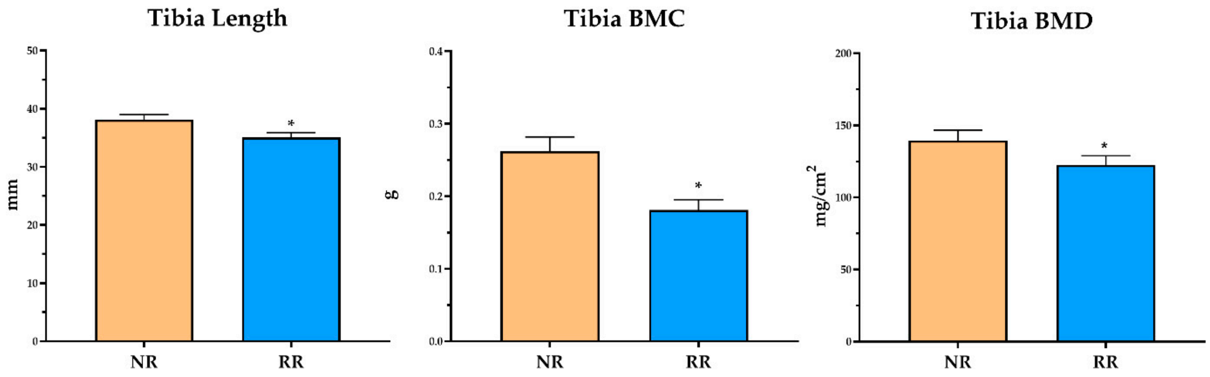

Figure S6. Effect of three-week 70% dietary intake on tibia length, and densitometry parameters. Data represented as mean ± SD. BMC, bone mineral content; BMD, bone mineral density; NR, non-restricted group; RR, restricted group. * P-value <0.05.

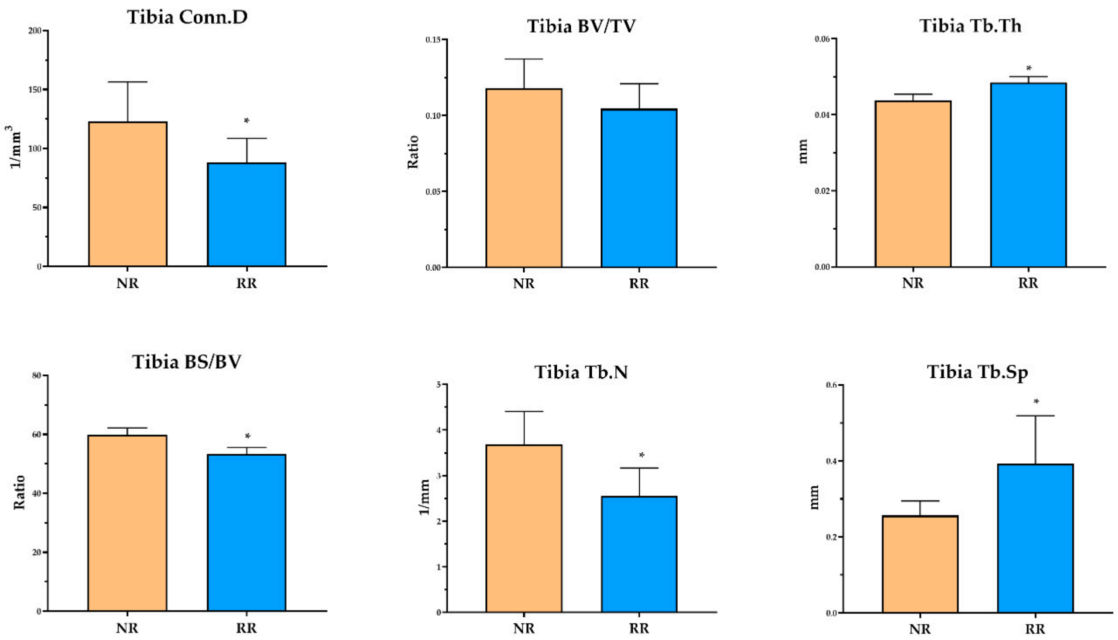

Figure S7. Effect of three-week 70% dietary intake on tibia trabecular microstructure. Data represented as mean ± SD. BS/BV, bone surface/volume fraction; BV/BT, bone volume fraction ; Conn. D., connective density; NR, non-restricted group; RR, restricted group; Tb.N., trabecular number; Tb.Sp., trabecular separation; Tb.Th., trabecular thickness. * P-value <0.05.

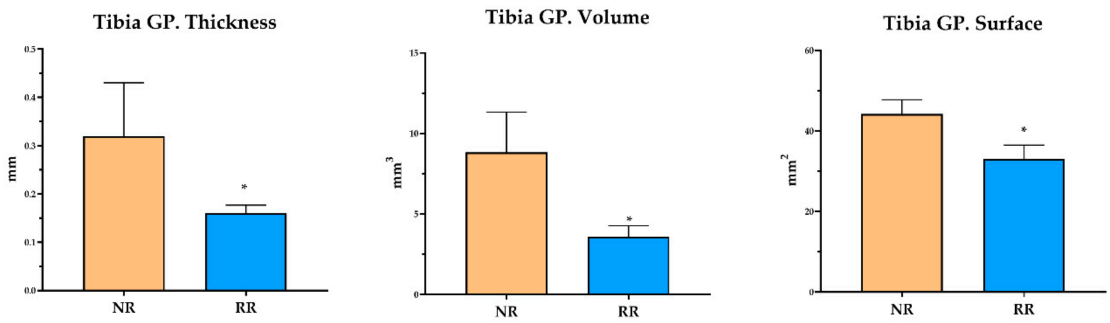

Figure S8. Effect of three-week 70% dietary intake on tibia growth plate. Data represented as mean ± SD. GP, Growth plate; NR, non-restricted group; RR, restricted group. * P-value <0.05.

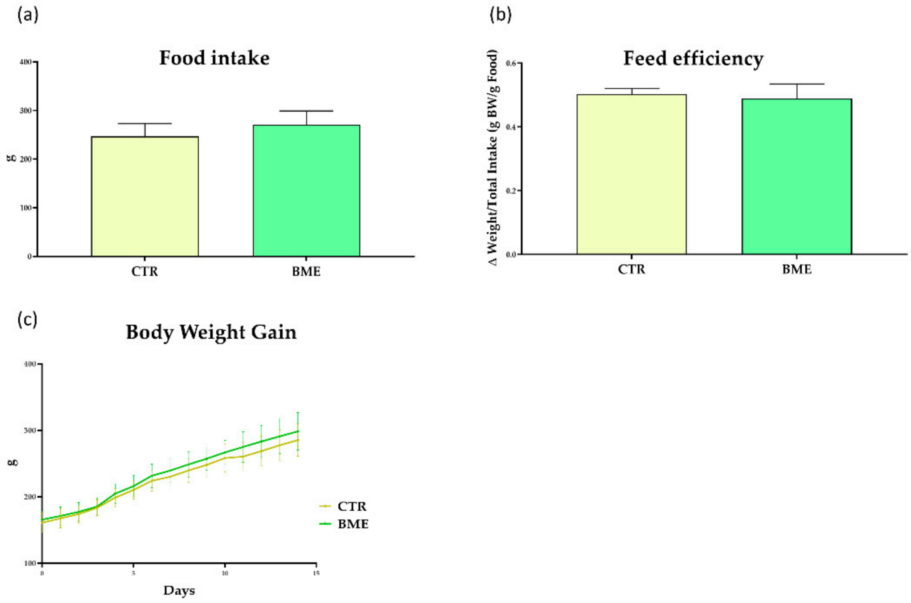

Figure S9. Food consumption and body weight gain during the two-week refeeding period. Data represented as mean ± SD. BW, body weight; BME, group refed with the diet supplemented with whey protein concentrate enriched in bovine milk exosomes; CTR, group refed with the control diet.
